# 

**Published:** 1854-01

**Authors:** 


Vol. VII. JANUARY, 1854. No. 2.

THE

### DENTAL REGISTER

OF

### THE WEST.

EDITED BY

JAMES TAYLOR, M. D., D. D. S.

PUBLISHED QUARTERLY

AT $2 PER ANNUM.

AGENTS.

J. B. DUNLEVY, Pittsburgh, Pa.
DRS. SPALDING & FITHIAN, St. Louis, Mo.
Messrs. JONES, WHITE & MCCURDY, Phila.
New York City, and Boston, Mass.
Dr. J. M. BROWN, Cincinnati.
SOLOMON BROWN, New York city.
JOHN KLEIN, Phil. Pa.

CINCINNATI:
PRINTED BY T. WRIGHTSON, 167 WALNUT STREET.
1854.

